# Defining molecular basis for longevity traits in natural yeast isolates

**DOI:** 10.1038/npjamd.2015.1

**Published:** 2015-09-28

**Authors:** Alaattin Kaya, Siming Ma, Brian Wasko, Mitchell Lee, Matt Kaeberlein, Vadim N Gladyshev

**Affiliations:** 1 Division of Genetics, Department of Medicine, Brigham and Women’s Hospital and Harvard Medical School, Boston, MA, USA; 2 Department of Pathology, University of Washington, Seattle, WA, USA

## Abstract

**Background:**

The budding yeast has served as a useful model organism in aging studies, leading to the identification of genetic determinants of longevity, many of which are conserved in higher eukaryotes. However, factors that promote longevity in a laboratory setting often have severe fitness disadvantages in the wild.

**Aims and Methods:**

To obtain an unbiased view on longevity regulation, we analyzed how a replicative lifespan is shaped by transcriptional, translational, metabolic, and morphological factors across 22 wild-type *Saccharomyces cerevisiae* isolates.

**Results:**

We observed significant differences in lifespan across these strains and found that their longevity is strongly associated with up-regulation of oxidative phosphorylation and respiration and down-regulation of amino- acid and nitrogen compound biosynthesis.

**Conclusions:**

As calorie restriction and TOR signaling also extend the lifespan by adjusting many of the identified pathways, the data suggest that the natural plasticity of yeast lifespan is shaped by the processes that not only do not impose cost on fitness, but also are amenable to dietary intervention.

## Introduction

The idea of slowing aging and extending lifespan of organisms has attracted much attention, leading to the identification of numerous factors that mitigate the effects of the aging process. At the cellular level, the driving force behind aging may be the inevitable accumulation of a myriad different forms of molecular damage.^1^ Many genetic and pharmacological interventions have been discovered that increase the lifespan of model organisms, including some with single-gene effects.^2,3^ In addition, diverse classes of genes have been reported to be involved in lifespan control, pointing to several key regulatory pathways. However, it remains to be seen whether similar strategies may be applied to combat aging in humans. A major challenge in the field is that many of the findings apply to model organisms in laboratory settings, but these longevity conditions may come at the expense of fitness, making them detrimental when organisms are in their natural environment.

Aging is a process that involves complex gene networks. While broad genome manipulation is not yet practical in higher eukaryotes, fine tuning these gene networks by environmental or dietary factors may offer a solution. It has been shown that manipulations such as calorie restriction (CR), oxygen availability, pH, and alternative carbon sources can modulate gene expression and the aging process.^4–7^ CR is among the most studied and widely used longevity interventions, which can extend lifespan in almost all model organisms.^2^ Although the precise mechanisms of CR-mediated lifespan extension remain debatable, it is known that CR causes a metabolic shift from fermentation to respiration in yeast and that mitochondrial metabolism tends to increase in multicellular eukaryotes subjected to CR.^8–10^ These findings also agree with the effects observed by manipulating various lifespan-regulating pathways, such as target of rapamycin (TOR) signaling.^11^ Suppression of TOR signaling mimics the reduction of nutritional input under CR in yeast and extends lifespan while concomitantly increasing mitochondrial respiration.^11,12^ Taken together, these studies link elevated mitochondrial function with lifespan, suggesting that a metabolic switch to oxidative metabolism is beneficial with regard to delaying aging.

The fact that metabolic pathways can be modulated by both CR and TOR inhibition suggests that complex processes such as aging may also be amenable to environmental and genetic manipulations. It is conceivable that the interaction between environmental factors and gene networks can explain the diverse phenotypes of species inhabiting different ecological niches. It is known that environmental adaptation and parallel evolution help create the genetic diversity for selection in natural populations.^13^ By evaluating the lifespan differences among natural populations of closely related strains or species, one may obtain insights into the underlying mechanisms that modulate aging and longevity. Toward this goal, in the current work we employed a powerful aging model, the budding yeast. Analyses of the aging process in *Saccharomyces cerevisiae* have mostly been performed on a small number of laboratory-adapted strains, but whether the identified mechanisms can explain the lifespan variation across natural strains is unknown. We evaluated the lifespans of 22 natural isolates of *S. cerevisiae* ^14^ and used transcriptome, proteome, metabolome, and morphology data^15^ to identify the signatures associated with natural lifespan variation. Our data suggest that increased replicative lifespan (RLS) in natural yeast populations is associated with increased oxidative phosphorylation and reduced amino-acid biosynthesis. Our study also represents a new approach that combines phenotypic variation across yeast populations with high-throughput data to elucidate underlying molecular mechanisms driving this variation.

## Materials and Methods

### Yeast strains

Diploid natural isolates of *S. cerevisiae* were obtained from the Sanger Institute and are summarized in Table 1. These strains are well characterized.^14,16^ The diploid laboratory strain BY4743 was purchased from American Type Culture Collection (ATCC).

### Replicative lifespan assay

For each strain, cells were freshly grown on yeast extract peptone dextrose plates before dissections. Several colonies were streaked onto new yeast extract peptone dextrose plates using pipette tips. After overnight growth, 40–50 dividing cells were lined up. Newborn daughter cells were chosen for RLS assays after the first division using a micromanipulator. Plates were incubated at 30 °C between dissections and left at 4 °C during night. All RLS assays were performed in standard yeast extract peptone dextrose plates with 2% glucose or 3% glycerol as previously described.^17^ For each natural isolate, at least two independent assays were performed. Each assay contained 20–40 mother cells.

### Phenotypic data

Growth rates were determined using a Bioscreen C MBR (http://www.bioscreen.fi/) machine by analysis of optical density in the OD_420–580_ range as previously described in combination with the YODA software package.^18^ The data on transcripts, peptides (proteins), metabolites, and morphology were downloaded from Yeast Resource Center (http://www.yeastrc.org/g2p/download.do). Values corresponding to the 22 strains were extracted; metabolite data were not available for 378604X. Metabolites with missing values in more than one strain (other than 378604X) were discarded; the remaining missing values (6 out of 107 metabolites) were imputed based on 10 nearest neighbors, using ‘knnImputation’ function of R package ‘DMwR’. For comparison across the phenotypic data, the values were standardized across the strain by setting mean=0 and s.d.=1. In addition, for genes represented by multiple peptides, we calculated the mean standardized values to perform the regression.

### Principal component analysis

Principal component analysis was performed on standardized values using R package ‘stats’.^19^ To identify the underlying pathways, the factors in each of the first three principal components (PCs) were ranked by their contributions, and pathway enrichment analysis was performed on the top 10% factors using DAVID (https://david.ncifcrf.gov/), after correcting for background.

### Phylogenetic regression by generalized least squares

Phylogenetic regression was performed by generalized least squares method using R packages ‘nmle’ and ‘phylolm’.^20,21^ Four models of trait evolution were tested: (i) complete absence of phylogenetic relationship (‘Null’); (ii) Brownian Motion model (‘BM’); (iii) BM transformed by Pagel’s lambda (‘Lambda’); and (iv) Ornstein–Uhlenbeck model (‘OU’). For Lambda and OU models, the parameters were estimated simultaneously with the coefficients using maximum likelihood. The best-fit model was selected based on maximum likelihood. Strength of correlation was based on the p-value of regression slope. To confirm robustness of the results, regression was performed by leaving out each strain, one at a time, and computing *P* values using the remaining strains.

### Relative coverage of mitochondrial DNA

Genomic reads of strains examined in our study were downloaded from Yeast Resource Center (http://www.yeastrc.org/g2p/download.do) and mapped to reference genome of *S. cerevisiae* strain S288c (http://www.yeastgenome.org/download-data/sequence). The average coverage per base across the chromosomes (excluding positions 45,000–50,000 of chromosome XII) was calculated using R package ‘ShortRead’ for each strain. The relative coverage of mitochondrial DNA was expressed as the normalized ratio of per-base coverage of mitochondrial DNA to normalized per-base coverage of chromosomes.

### Differential expression between long-lived and short-lived groups

Six closely related strains were grouped into long lived (YJM981, YJM975, and DBVPG1373) and short lived (YJM978, NCY361, and YS2). Differential expressions of the phenotypic data were calculated using R package ‘limma’.^19^

### Mitochondrial protein expression

Logarithmically growing cells (5 ml and OD_600_=0.6) were harvested and incubated in 150 μl extraction buffer (1.85 mmol/l NaOH and 2% β-mercaptoethanol) on ice for 10 min. Then, 150 μl of 50% trichloroacetic acid was added and incubated for 30 min on ice. After incubation, the cells were pelleted and supernatant aspirated. After 30 min of air drying, the pellets were heated at 60 °C in sodium dodecyl sulfate loading buffer and 4 μl of each sample was analyzed by sodium dodecyl sulfate–polyacrylamide gel electrophoresis. To examine the expression of mitochondrial proteins, western blotting was carried out with antibodies against mitochondrial outer membrane protein Por1 (Abcam, Cambridge, MA, USA, cat:ab110326). The membranes were stripped and developed with antibodies against phosphoglycerate kinase (Pgk1; Life Technologies, Grand Island, NY, USA, cat:459250) as an internal loading control.

## Results

### Variation in replicative lifespan across natural yeast isolates

**Figure 1:**
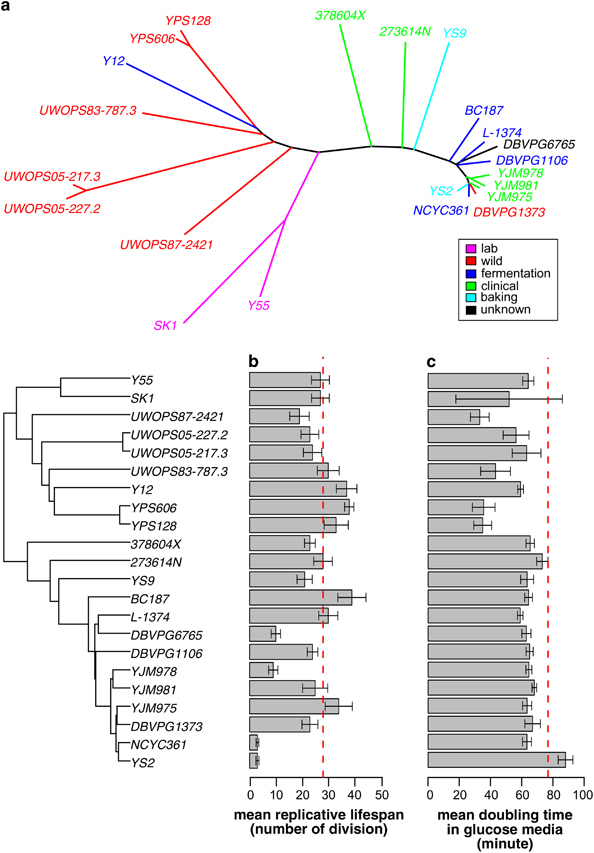
Yeast strains examined in this study. (**a**) Phylogenetic relationship. The tree was constructed based on the alignment of complete genome sequences of the strains, using MEGA 6.06^49^ and neighbor joining method.^50^ The branches are colored according to strain types shown in the legend in the lower right corner. (**b**) Mean replicative lifespan and (**c**) mean growth rate (doubling time) of the strains in glucose media. The strains are ordered by phylogeny. The error bars indicate s.e. Red dotted lines indicate the mean replicative lifespan (**b**) and doubling time (**c**) of the reference strain BY4743.

Phylogenetic analysis using complete genome sequence alignment of 22 natural *S. cerevisiae* isolates revealed a complex cladogram that could be divided into two main groups (Figure 1). Assaying these isolates at 30 °C on standard yeast extract peptone dextrose plates, we observed over 10-fold variation in RLS (Pearson correlation coefficient=0.95 between mean and maximum lifespans; Figure 1b and Table 1). BC187 showed the largest number of cell divisions (mean=39; maximum=60); NCYC361 and YS2 had the fewest (mean=3 for both; maximum=7 for NCYC361 and 9 for YS2); and many strains produced on average 20–30 daughter cells, similar to BY4743, a standard laboratory diploid strain and the parental strain of the yeast open reading frame deletion collection (Table 1).

### Growth of wild-type isolates in liquid culture

Changes in growth rate have previously been shown to affect mRNA, protein, and metabolite levels,^22–24^ and a recent study has reported a positive correlation between time spent in the G1 phase of the cell cycle and RLS in the yeast.^25^ To determine a potential relationship between growth rate and lifespan, we monitored the growth of these strains by automated Bioscreen-C growth analyzer and calculated the doubling time in both glucose and glycerol medium. Of the 22 isolates, 21 grew faster than BY4743 strain, and four strains doubled in <50 min (Figure 1c, Table 1) in glucose medium. However, we found only a weak negative correlation between the doubling time and mean lifespan (Pearson correlation coefficient=−0.42). In addition, we observed that all strains can utilize glycerol as a carbon source, which indicates these strains are capable of mitochondrial respiratory metabolism (Table 1).

### Phenotypic variation across strains

Gene expression, proteomic, metabolomic, and morphological data for these 22 strains have been reported previously.^15^ After our filtering and quality control, the data set contained RNA-seq reads for 6,207 transcripts; proteomic measurement of 6,842 peptide fragments corresponding to 1,643 unique genes; mass spectrometric quantification of 107 metabolites; and quantitative microscopy of 392 morphological phenotypes (Materials and Methods). In particular, 1,641 unique genes were represented by both transcripts and peptides, but the correlation between the transcript and protein levels was not strong (median Spearman correlation coefficient=0.31; Supplementary Figure 1A). Similar conclusions were reached when we used the mean peptide values for each gene instead (median Spearman correlation coefficient=0.28; Supplementary Figure 1B).

**Figure 2:**
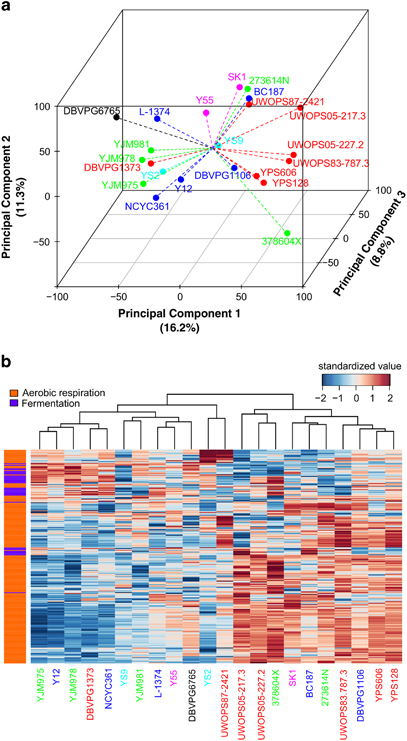
Phenotypic variation across the strains. (**a**) Principal component analysis (PCA) of combined data. PCA was performed by combining transcripts, proteins (peptides), and morphology data (metabolite data were not available for strain 378604X and were omitted). Percentage variance explained by each principal component (PC) is shown in the parentheses. The strains are colored using the same scheme as Figure 1a. See Supplementary Figure 2 for separate PCA plots on each class of phenotype data and for cumulative percentage of variance explained by the PCs. (**b**) Relative levels of transcripts and proteins involved in aerobic respiration or fermentation. The heat map shows the transcripts and proteins with top contribution to PC 1 and involved in aerobic respiration or fermentation (Supplementary Table 1). Hierarchical clustering was performed using complete linkage.

To visualize phenotypic variation across these strains, we performed PC analysis on each type of the phenotypic data as well as on the combined data (Figure 2a, Supplementary Figure 2A–D; the combined data excluded metabolites as values were not available for strain 378604X). The observed patterns resembled the phylogenetic relationship, with the first three PCs explaining 36–53% of total variance (Supplementary Figure 2E). Examination of the genes contributing to the first three PCs in the combined data revealed a distinctive set of Gene Ontology (GO; http://geneontology.org/) terms and Kyoto Encyclopedia of Genes and Genomes (KEGG; http://www.genome.jp/kegg/) pathways, including oxidative phosphorylation (PC1), aerobic respiration (PC1), mitochondrion (PC1), response to temperature stimulus (PC1), ribosome (PC2), protein synthesis (PC2), regulation of translation (PC2 and PC3), ribonucleoprotein complex (PC3), and ribosome biogenesis (PC3) (Supplementary Table 1). These results suggest that the strains predominantly differ in energy metabolism, protein synthesis, and ribosome regulation. Consistent with a previous report,^15^ along PC1 the strains segregated largely according to their relative preferences for aerobic respiration or fermentation (Figure 2b).

### Correlation between phenotype and lifespan

To identify a link between phenotypic variation and lifespan, we performed phylogenetic regression by generalized least squares, uncovering the phenotypes associated with longevity after accounting for the phylogenetic relationship of these strains.^26–28^ Regression was performed between phenotypic values and any one of the following lifespan measurements: mean RLS, maximum RLS (Max RLS), log mean RLS, and log maximum RLS. Different models of trait evolution were tested and the best-fit model was then selected based on maximal likelihood (Materials and Methods). To assess robustness of the relationship, we also left out one yeast strain at a time and recalculated regression slopes using the remaining strains. This ensured the overall relationship did not depend on a particular isolate.

The four different RLS measurements yielded very similar results, with Pearson correlation coefficients ranging between 0.90 and 0.98 for the regression slopes. We defined the top hits as phenotypes with statistically significant regression slopes under at least two different RLS measures, and identified 249 gene transcripts, 347 peptide fragments (representing 216 unique genes), 5 metabolites, and 43 morphology features (Supplementary Table 2). Among the top gene transcripts and protein fragments, only 10 unique genes were supported by both measures (Supplementary Figure 3), consistent with the weak correlation between transcript and protein levels noted above (Supplementary Figure 1). When the mean protein values were used for calculation, 88 genes reached statistical significance, 80 of which were also supported based on protein fragments (Supplementary Figure 3, Supplementary Table 2).

**Figure 3:**
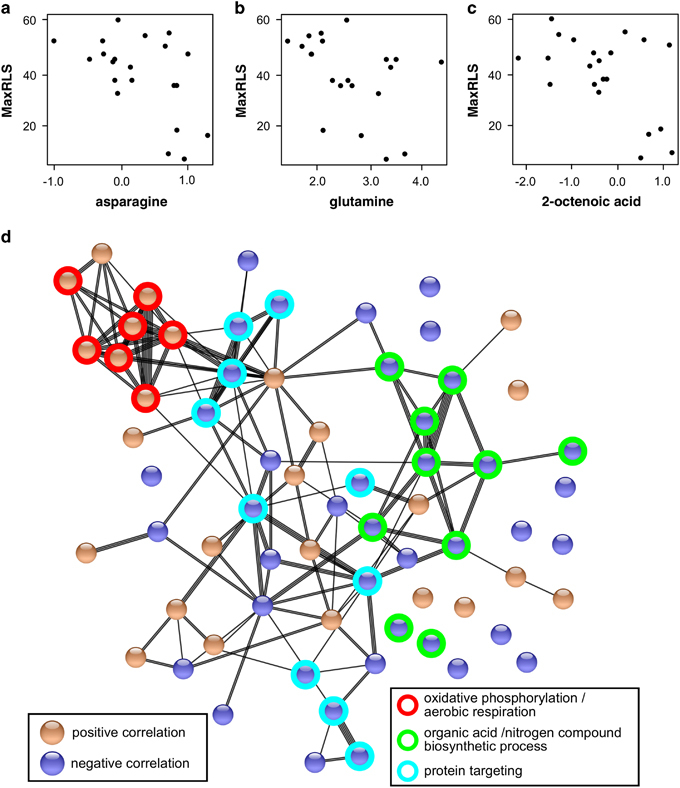
Selected phenotypes correlating with replicative lifespan. Levels of (**a**) asparagine, (**b**) glutamine, and (**c**) 2-octenoic acid negatively correlate with maximum replicative lifespan (Max RLS). Regression slope *P* values: (**a**) 0.014; (**b**) 0.042; and (**c**) 0.019. (**d**) Protein–protein interaction network of the top hits identified by the mean protein values. The interaction network is based on STRING database (evidence view). Genes without interacting partners are omitted. Selected pathways are indicated by colored rings. Most of the proteins here showed significant correlation to all four RLS measures. See Supplementary Table 2 for more details.

With regard to morphology measures, features such as ‘maximal intensity of nuclear brightness divided by average’, ‘nucleus roundness in mother cell’, and ‘length from bud tip to mother cell’s short axis on nucleus C’ showed significant negative correlation with RLS, whereas ‘fitness in nucleus C’ correlated positively with longevity (see Saccharomyces Cerevisiae Morphological Database (http://scmd.gi.k.u-tokyo.ac.jp/datamine/ParameterHelp.do) for detailed descriptions of the parameters). Among the metabolite top hits, asparagine showed negative correlation with Max RLS (*P* value=0.014) and Log Max RLS (*P* value=0.017; Figure 3a). A related amino acid, glutamine, also negatively correlated with Max RLS (*P* value=0.042) and weakly with Log Max RLS (*P* value=0.055; Figure 3b). This was of note, as the TOR pathway is known to be regulated by the levels of amino acids, especially intracellular glutamine.^29^ Treating yeast cells with methionine sulfoximine, an inhibitor of glutamine synthetase, has been shown to decrease both intracellular glutamine levels and TOR-dependent signaling^30^ while increasing RLS,^31^ whereas removal of either asparagine or glutamate from the medium produced a dose-dependent effect on chronological lifespan^32^ (chronological lifespan is the survival time of populations of non-dividing cells, while RLS is the number of daughter cells produced by a mother cell before senescence; they are related but not identical). We also found 2-octenoic acid to correlate negatively with Max RLS (*P* value =0.019) and Log Max RLS (*P* value =0.014; Figure 3c). This compound is known to be elevated in mitochondria, but its effect on aging is not known. Some of the transcript and protein top hits have also been implicated in lifespan regulation in yeast. For example, the protein levels of ADH1p (alcohol dehydrogenase, coded by *YOL086C*) correlated negatively with both mean RLS and Max RLS, and deletion of *ADH1* was found to extend RLS by 23% in MATα and 15% in MATa (ref. 33). *DCW1* (also known as *YKL046C*, coding for a putative mannosidase in cell wall biosynthesis), whose transcript levels correlated negatively with all four RLS measurements, was previously identified in a genetic screen to increase yeast chronological lifespan when deleted.^34^ In addition, a number of top hits correlating positively with longevity at the transcript (e.g., *VRP1* (*YLR337C*), *KGD1* (*YIL125W*)) and protein (e.g., PET9p (YBL030Cp), SP160p (YJL080Cp), GSY2p (YLR258Wp)) levels were previously shown to decrease RLS or chronological lifespan when deleted or mutated.^33,35–37^

### Networks and pathways represented by top hits

To further understand the biological pathways underlying natural regulation of lifespan, we performed pathway enrichment analysis using DAVID^38^ (Supplementary Table 3). The enrichment results for the protein fragments were especially significant. Among the protein fragments correlating positively with longevity, the enriched terms included ‘oxidative phosphorylation’, ‘mitochondrial respiratory chain’, ‘ion transport’, ‘hexose metabolic process’, ‘glucose metabolic process’, and ‘aerobic respiration’. On the other hand, for those correlating negatively with longevity, ‘amino-acid biosynthesis’, ‘organic acid biosynthetic process’, ‘nitrogen compound biosynthetic process’, ‘nucleotide binding’, ‘cofactor binding’, and ‘glycolysis’ were enriched. Many of these terms were similarly enriched when we carried out calculations using the mean protein values (Supplementary Table 3). In comparison, the enrichment statistics were weaker for the transcripts, even though the numbers of top hits were similar. Among those with positive correlation, enrichment was observed for ‘ion transport’, ‘mitochondrial membrane part’, ‘ATP biosynthetic process’, ‘oxidative phosphorylation’, and ‘actin binding’. For the transcripts with negative correlation to lifespan, the enriched terms included ‘RNA polymerase II transcription factor activity’, ‘transcription regulator activity’, ‘microtubule’, ‘regulation of RNA metabolic process’, and ‘mRNA splicing’. Overall, the results suggest that the long-lived strains tend to upregulate oxidative phosphorylation, aerobic respiration, and ion transport, and downregulate transcription, splicing, and various biosynthetic processes (especially amino-acid metabolism).

We visualized protein–protein interactions among the top hits using STRING^39^ and found the network is significantly enriched in interactions. The top hits identified using the mean protein values were grouped into several prominent clusters, including oxidative phosphorylation and aerobic respiration (positive correlation); organic acid and nitrogen compound biosynthetic process (negative correlation); and protein targeting (negative correlation) (Figure 3d). Similar clusters of the top hits were observed for transcripts and protein fragments data (Supplementary Figure 4), suggesting that the top hits, rather than being a random collection of genes, represent interconnected nodes in regulatory networks and pathways.

### Mitochondrial abundance and composition of the strains

As the results suggested a relative upregulation of oxidative phosphorylation and aerobic respiration among the long-lived strains, we examined more closely the nature of such differences. First, the genomic reads of these strains^15^ were used to calculate average coverage of the mitochondrial DNA relative to that of the nuclear DNA (Table 1, Supplementary Figure 5A), as a proxy for mitochondria copy number. Although the relative coverage was highest in YJM381 (8.0) and lowest in YPS128 (3.0), the values were relatively constant for most of the strains (4.0–5.0) and there was no overall correlation with longevity (Pearson correlation *P* value=0.31 with Max RLS and 0.53 with mean RLS). Moreover, western blotting confirmed the similar expression of a mitochondrial marker protein Por1 in these strains (Supplementary Figure 5B). In addition, the doubling times in glycerol media were similar (100–120 min for most of the strains, with exception of >180 min for YS2, DBVPG1373, and Y55 strains; Supplementary Figure 5C, Table 1), suggesting the longevity variation across these strains could not be simply explained by total mitochondrial content or number.

**Figure 4:**
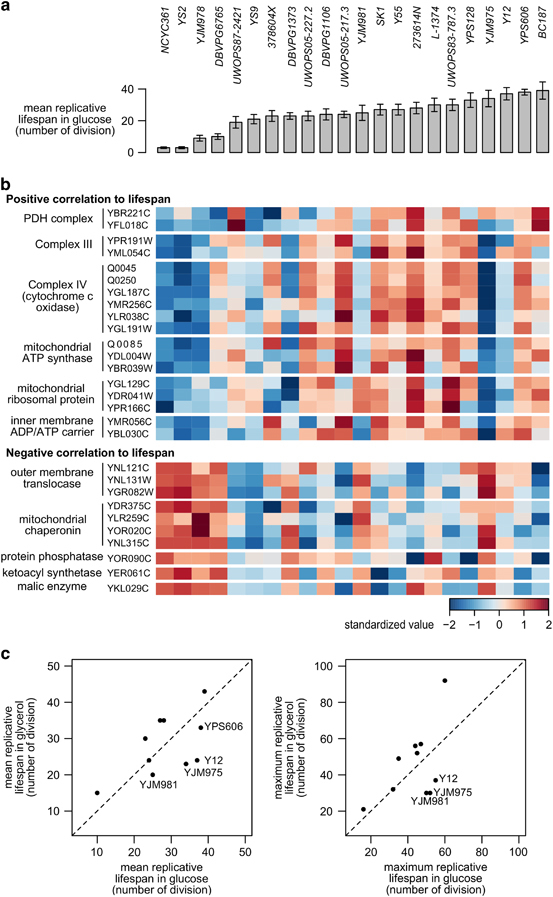
Mitochondrial respiratory composition varies across the strains according to lifespan. (**a**) Mean replicative lifespan of strains. Strains are ordered according to their mean lifespan (see also Figure 1b). (**b**) Levels of certain proteins correlate with lifespan. The mean values of the selected proteins (related to mitochondrial function) are shown. For each protein, the levels were standardized by setting mean=0 and s.d.=1 across the strains. The patterns of DBPVG1373, YJM981, YJM975, and Y12 strains showed different patterns. See Supplementary Table 2 for more detail. (**c**) Effect of growth on a respiratory substrate on lifespan. Replicative lifespan of 10 strains was tested on yeast peptone glycerol (3% YPG) plates and expressed as mean (left) and maximum (right) replicative lifespan. Except for the three long-lived outlier strains (YJM981, YJM975, and Y12), all strains either increased or did not change lifespan when their growth substrate was switched from glucose to glycerol.

However, when we examined the top hits based on mean protein values (Supplementary Table 2), a trend emerged. Approximately one-third of these proteins were related to mitochondria, with characteristic distribution patterns across the strains depending on their lifespan (Figure 4b). For example, the longer-lived strains generally had higher levels of proteins belonging to pyruvate dehydrogenase complex, complex III, complex IV, mitochondrial ATP synthase, inner membrane ADP/ATP carrier, as well as mitochondrial ribosomal proteins. On the other hand, long-lived strains had lower relative levels of outer membrane translocases, mitochondrial chaperonins, and certain metabolic enzymes. The results suggest that the mitochondrial metabolism may vary widely across the strains according to their longevity. The longer-lived strains seem to enhance the electron transport chain and oxidative phosphorylation capacity, whereas the shorter-lived strains place more emphasis on protein folding and outer membrane transport. Although the biological implications underlying these observations need to be further explored, the results show that distinct mitochondrial composition is associated with different yeast strains, and such patterns agree well with the observed lifespan variation.

### Comparison of related long-lived and short-lived strains

A number of our strains (YJM978, YJM981, YJM975, DBVPG1373, NCYC361, and YS2) are closely related to each other phylogentically (Figure 1a), but differ significantly in replicative lifespan (Figures 1b and 4a). In particular, they may be grouped into long lived (YJM981, YJM975, and DBVPG1373) and short lived (YJM978, NCY361, and YS2). If our findings above were valid, then we should observe similar sets of genes and pathways differentially expressed between these two groups. The analysis showed that the genes involved in ‘hexose metabolic process’, ‘glucose metabolic process’, and ‘glycolysis’ were expressed highly in the long-lived strains, whereas those involved in ‘organic acid biosynthetic process’, ‘amino-acid biosynthesis’, and ‘cofactor binding’ were expressed at relatively low levels (Supplementary Table 4). Compared with the pathways we identified above, the genes involved in oxidative phosphorylation and aerobic respiration did not emerge as top hits, and there were not as many proteins related to mitochondria among these six strains. This is likely because all of these strains prefer fermentation over aerobic respiration (Figure 2b), and they already share similar mitochondrial composition profiles (Figure 4b). Among the strains designated as YJM are clinical isolates and their adaptation to longevity appears to be different from other strains. For example, YJM975 and YJM981 are long lived, but their mitochondrial patterns are similar to the short-lived strains. Perhaps, their longevity is based on lineage-specific features that are not shared by other long-lived isolates. We found that these outlier strains showed a decreased lifespan when grown on glycerol, whereas most other strains increased lifespan under these conditions (Figure 4c). Importantly, deviation in the expression of mitochondrial proteins from the overall pattern (Figure 4b) agreed well with the capacity of a respiratory substrate to increase lifespan (Figure 4c). Nevertheless, among the long-lived strains we observed lower levels of expression of genes and proteins involved in biosynthetic processes (most of which were cytoplasmic, Supplementary Table 4), in agreement with our observations based on the 22 strains. This suggests that long lifespan can also arise without substantially altering the mitochondrial composition, although the reduction in biosynthesis seems to be a common feature.

## Discussion

Availability of high-quality genome sequence of *S. cerevisiae* has made yeast an attractive model for dissecting complex traits associated with various phenotypes. Comparative genomics across multiple natural yeast isolates enabled the identification of extensive natural genetic variation at the single nucleotide polymorphism level and the elucidation of genotype to phenotype relation in several traits.^40^ Here we ask: can similar strategies be applied to understand the common determinants of aging and longevity?

Using high throughput omics data, we examined 22 yeast natural isolates, which were found to vary over 10-fold in RLS. These isolates occupy diverse ecological niches and face different evolutionary pressures, so their natural lifespan variation must be encoded in their respective genomes. However, it has been challenging to characterize the cumulative effect of multiple alleles on a phenotype, especially if the underlying process involves a complex gene network. Alternatively, one may look at variation in gene transcripts (transcriptome) and gene products (proteome) and correlate them and the associated pathways with the phenotypic traits, as the genotypic variation should be reflected in the expression variation in order to create the associated phenotypic differences.^41^

To identify a link between transcript variation and lifespan, we performed phylogenetic regression and identified genes correlating with RLS, some of which were previously implicated in longevity regulation. Our pathway analysis showed that the long-lived strains tend to upregulate oxidative phosphorylation, aerobic respiration, and ion transport, and downregulate transcription, splicing, glycolysis, and various biosynthetic processes, most notably amino-acid synthesis. In particular, the variation in mitochondrial respiratory composition of these strains agrees well with their differences in lifespan when grown on glucose and glycerol. Mitochondria are at the heart of cellular metabolism and energy production, and increased mitochondrial respiratory capacity has been linked to longevity.^12,42^ It was observed in *tor1* null yeast strain that lifespan extension was accompanied by increased mitochondrial respiration (particularly oxidative phosphorylation complex subunits) without increased mitochondrial biogenesis during growth on glucose.^12,42^ Upregulation of respiration increases mitochondrial membrane potential and reactive oxygen species production, which may act as adaptive signals to induce stress resistance and extend lifespan.^43,44^ It is also possible that many of these natural isolates reside in environments with low fermentable carbon sources, so that they undergo diauxic shift and metabolize respiratory carbon sources. Shifting from fermentable (glucose) to respiratory carbon sources is also known to extend both replicative and chronological lifespan in yeast.^45^

Genetic variation responsible for lifespan differences may also affect metabolite levels and morphology. Among the examined metabolites, glutamine and asparagine showed strong negative correlation with RLS, which is consistent with the known inhibition of TOR activity and extension of chronological lifespan by removing glutamine or asparagine from yeast media^32^ and extension of RLS by treating cells with methionine sulfoximine.^31^ In terms of cell morphology, a number of nuclear features such as brightness, roundness, and distance to bud tip showed significant negative correlation with RLS, whereas ‘fitness in nucleus C’ correlated positively with longevity. Interestingly, longer-lived strains tend to possess smaller mother cell volume (Supplementary Figure 6), indicative of a potential compromise between mother cell size and lifespan, as has been previously observed for long-lived cells treated with ibuprofen.^25^ In agreement, inverse correlation between cell size and lifespan has been observed in yeast previously.^46^ Thus, here too, natural changes in a phenotype can be linked with longevity interventions and maybe used as aging biomarkers.

It should be noted that our method is limited to identifying the genetic and metabolic processes that show concerted changes in relation to longevity across these 22 strains, which are more likely to be generalizable and do not depend on single or a few strains. On the other hand, an exceptionally long-lived or short-lived strain can also have arisen due to certain strain-specific changes that are not shared by other isolates, and such changes may be missed by our method. Comparison among the six related long-lived and short-lived strains suggests there may be more than one way to achieve long lifespan, and it will be useful to examine strains across different evolutionary distances to identify the common features.

To our knowledge, this is the first report that analyzes inter-strain natural diversity of RLS at the population level using high throughput data. Natural isolates occupying diverse ecological niches may face different selection pressures and have evolved to adjust their gene expression, metabolism, longevity, and reproduction to ensure survival and propagation.^47^ Although evolution can sometimes provide different solutions to the same challenge,^48^ our data suggest a consistent set of genes and pathways are responsible for modulating the lifespan trait across a broad diversity of wild yeast isolates.

Finally, it has been unclear whether the previous findings of various longevity regulator genes identified in the laboratory setting could be translated to the natural environment. A possibility is that these lifespan-extending interventions may come at the expense of fitness. For example, many longest-lived *Caenorhabditis* *elegans* laboratory mutants tend to develop and move slowly and often show reduced fecundity, so they will probably be eliminated quickly for lack of competitive advantage in the wild. In addition, two-third of long-lived single gene–deletion mutants in yeast demonstrated significantly reduced fitness compared with isogenic wild-type cells.^45^ Our results show that natural changes in lifespan need not come at the expense of significant changes in fitness. Although it is difficult to compare fitness across the strains, the longer-lived yeast isolates are presumably well adapted to their respective ecological niches. This notion also agrees with the finding that one-third of single gene–deletion mutants in yeast showed no obvious changes in fitness.^45^ It should also be pointed out that our analysis is unbiased with regard to the genes and pathways involved in lifespan control and supports a possibility that multiple correlates cumulatively contribute to the longevity phenotypes. Specifically, we found that the ability of yeast cells to rely more heavily on respiration and repress their anabolic programs, even under conditions of glucose excess, are among the key adaptations that lead to increased lifespan. Importantly, as CR and TOR signaling are also known to extend lifespan by activating respiration and inhibiting biosynthetic processes, these data show that natural plasticity of yeast lifespan is shaped by pathways that both impose little cost on fitness and are amenable to dietary intervention. Thus, environment may be a trigger for changes associated with increased lifespan that are then fixed in the genomes.

## Figures and Tables

**Table 1:** Strains used in this study

*Strain name*	*Strain type*	*Source*	*Replicative lifespan*			*Doubling time in glucose (min)*		*Doubling time in glycerol (min)*		*Relative coverage of mitochondrial DNA*	*Cell size (μm)*
			*Maximum*	*Mean*	*s.e.*	*Mean*	*s.e.*	*Mean*	*s.e.*		
Y55	Lab	Grape	47	27	3.43	63.79	3.56	110.31	1.13	4.46	10
SK1	Lab	Soil	45	27	3.39	51.62	33.95	189.60	11.74	5.36	13
UWOPS87-2421	Wild	Plant	35	19	3.72	32.91	5.96	107.01	0.74	4.16	12
UWOPS05-227.2	Wild	Bee	37	23	3.39	56.03	8.23	116.59	4.93	4.41	11
UWOPS05-217.3	Wild	Plant	42	24	3.51	62.77	9.14	115.71	5.52	4.41	11
UWOPS83-787.3	Wild	Fruit	52	30	4.16	42.96	9.47	106.65	1.46	4.49	10
Y12	Fermentation	Palm wine	55	37	3.92	58.91	1.83	117.73	3.92	4.79	10
YPS606	Wild	Oak tree	44	38	1.76	35.43	7.22	98.50	1.29	3.85	10
YPS128	Wild	Oak tree	54	33	4.61	34.78	5.68	97.67	1.83	3.02	10
378604X	Clinical	Sputum	31	23	2.06	65.04	2.64	204.72	10.88	4.42	12
273614N	Clinical	Fecal	45	28	3.55	72.75	3.63	109.31	1.24	5.60	13
YS9	Baking	Unknown	37	21	2.90	63.08	4.15	131.35	0.91	3.29	14
BC187	Fermentation	Barrel	60	39	5.39	64.03	2.46	117.93	0.35	7.38	10
L-1374	Fermentation	Must	47	30	3.63	58.68	1.75	104.74	1.34	4.99	12
DBVPG6765	Unknown	Unknown	16	10	1.76	62.60	2.91	99.16	5.06	4.00	13
DBVPG1106	Fermentation	Grapes	32	24	2.00	64.70	2.26	109.41	1.52	5.46	12
YJM978	Clinical	Vaginal	18	9	1.76	64.20	1.89	110.30	4.21	5.75	12
YJM981	Clinical	Vaginal	50	25	4.78	67.68	1.43	102.18	3.16	7.98	14
YJM975	Clinical	Vaginal	52	34	5.18	63.07	2.89	106.81	3.28	5.95	11
DBVPG1373	Wild	Soil	35	23	3.02	66.58	4.93	123.39	2.48	4.87	11
NCYC361	Fermentation	Wort	7	3	0.53	62.97	2.84	117.85	0.75	5.19	12
YS2	Baking	Unknown	9	3	0.57	87.55	4.67	216.05	2.31	4.24	11
BY4743	Lab	Grape	44	28	1.67	76.38	2.89	163.62	7.17	4.46	NA

Natural isolates of yeast strains are shown along with their environmental niche, mean and maximum lifespan, maximum doubling time in glucose and glycerol media, relative coverage of mitochondrial DNA, and cell size.
