# Supplementary material for: Defining molecular basis for longevity traits in natural yeast isolates
Source: NPJ Aging Mech Dis. 2015 Sep 28;1:15001–. doi: 10.1038/npjamd.2015.1 (PMC4807016; doi:10.1038/npjamd.2015.1)
Supplement: Supplementary Information [file npjamd20151-s1.pdf]

Supplementary Table 1. Pathway enrichment analysis of genes with the top 10% contribution to the first three Principal Components (PCs). Enrichment analysis was performed using DAVID on the genes represented by transcripts and peptide fragments with top 10% contribution to the first three PCs in Figure 2A.

Supplementary Table 2. Phenotypes with significant correlation to mean replicative lifespan (Mean RLS), maximum replicative lifespan (Max RLS), log mean replicative lifespan (Log Mean RLS), and log maximum replicative lifespan (Log Max RLS). Only phenotypes with significant correlation to at least two of the RLS measurements are shown. “Best.Model” indicates the best-fit regression model; “Slope.coefficient” indicates the regression slope under the best-fit model (positive value indicates positive correlation; negative value indicates negative correlation); “p.value” indicates the regression slope p-value; “RobustTest.Min”, “RobustTest.Median”, and “RobustTest.Max” indicate the minimal, median, and maximal p-values when the regression is performed by leaving out one strain at a time. “peptide_mean” refers to the mean peptide values across all the peptide fragments for the unique genes. The “Note” column in “peptide_mean” indicates the number of peptide fragments for each unique gene.

Supplementary Table 3. Pathway enrichment analysis of the top hits. Enrichment analysis was performed using DAVID on the top hit genes indentified based on transcripts, peptide fragments, and mean peptide values (Supplementary Table 2). Analysis was performed separately for positive and negative correlations.

Supplementary Table 4. Phenotypic data differentially expressed between long-lived and short-lived groups. YJM981, YJM975, and DBVPG1373 were considered long-lived; YJM978, NCY361, and YS2 were considered short-lived. Differential expression analysis was performed using R package “limma”. Enrichment analysis was performed using DAVID on top hit genes indentified based on transcripts, peptide fragments, and mean peptide values.

SUPPLEMENTARY FIGURE 1

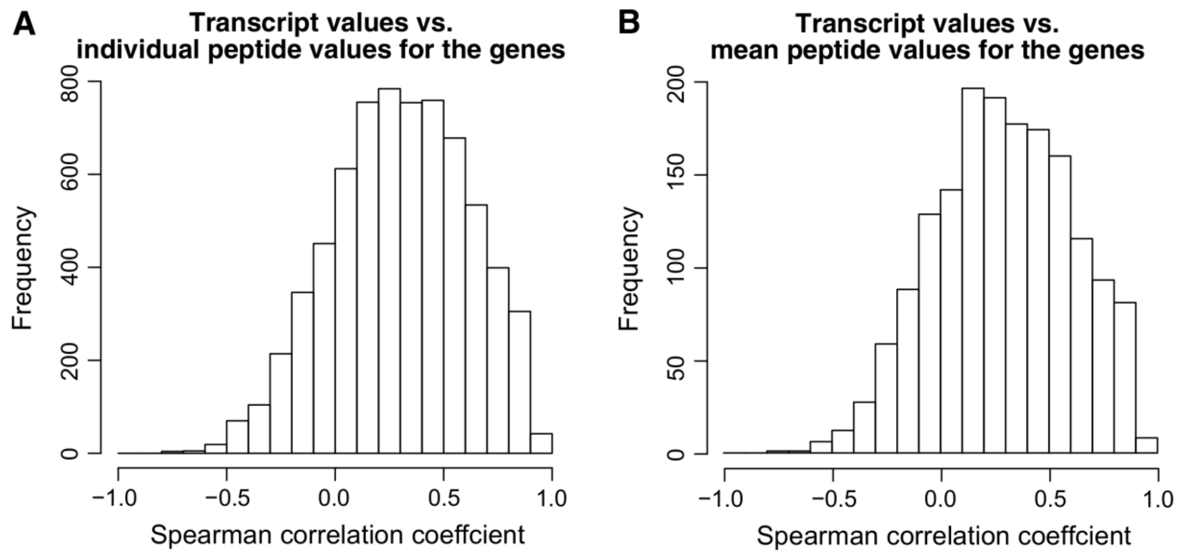

Supplementary Figure 1. Spearman correlation coefficients between transcript values and (A) individual peptide values, or (B) mean peptide values for genes. The 25th, 50th, and 75th percentile values are: (A) 0.08, 0.31, 0.54, respectively; and (B) 0.05,0.25,0.51, respectively.

SUPPLEMENTARY FIGURE 2

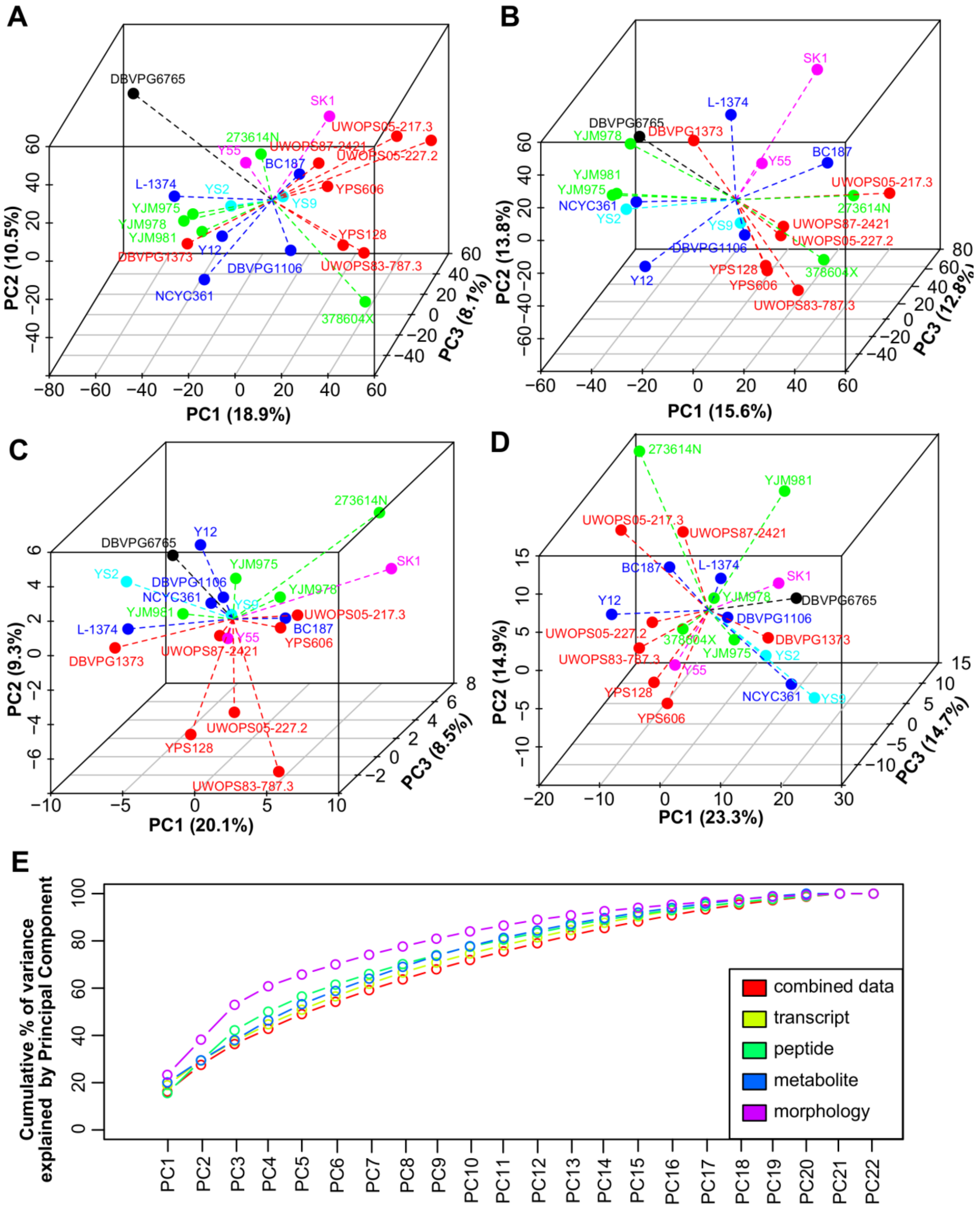

Supplementary Figure 2. Principal Component Analysis of (A) transcripts, (B) peptides, (C) metabolites, and (D) morphology data. The same color scheme was used as in Figure

1A. Percentage variance explained by each Principal Component (PC) is shown in parentheses. **(E) Cumulated percentage of variance explained by Principal Components.** Combined data: Figure 2A; transcripts, peptides, metabolites, and morphology: Supplementary Figure 2A-D, respectively.

SUPPLEMENTARY FIGURE 3

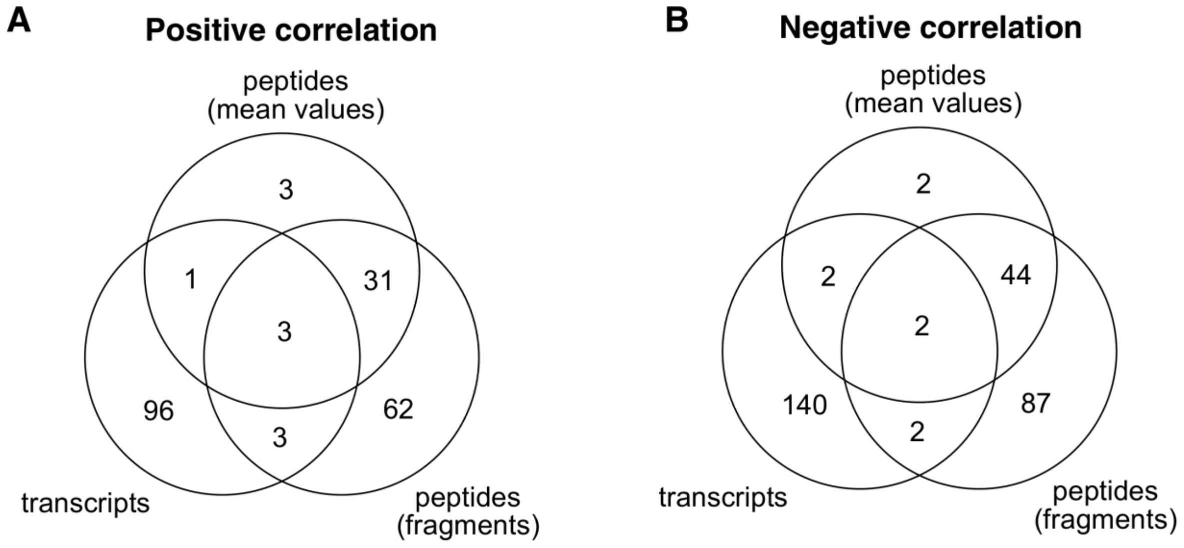

Supplementary Figure 3. Overlap of top unique genes identified based on transcripts, peptide fragments, and mean peptide values. Positive (A) and negative (B) correlation with lifespan is shown separately.

SUPPLEMENTARY FIGURE 4

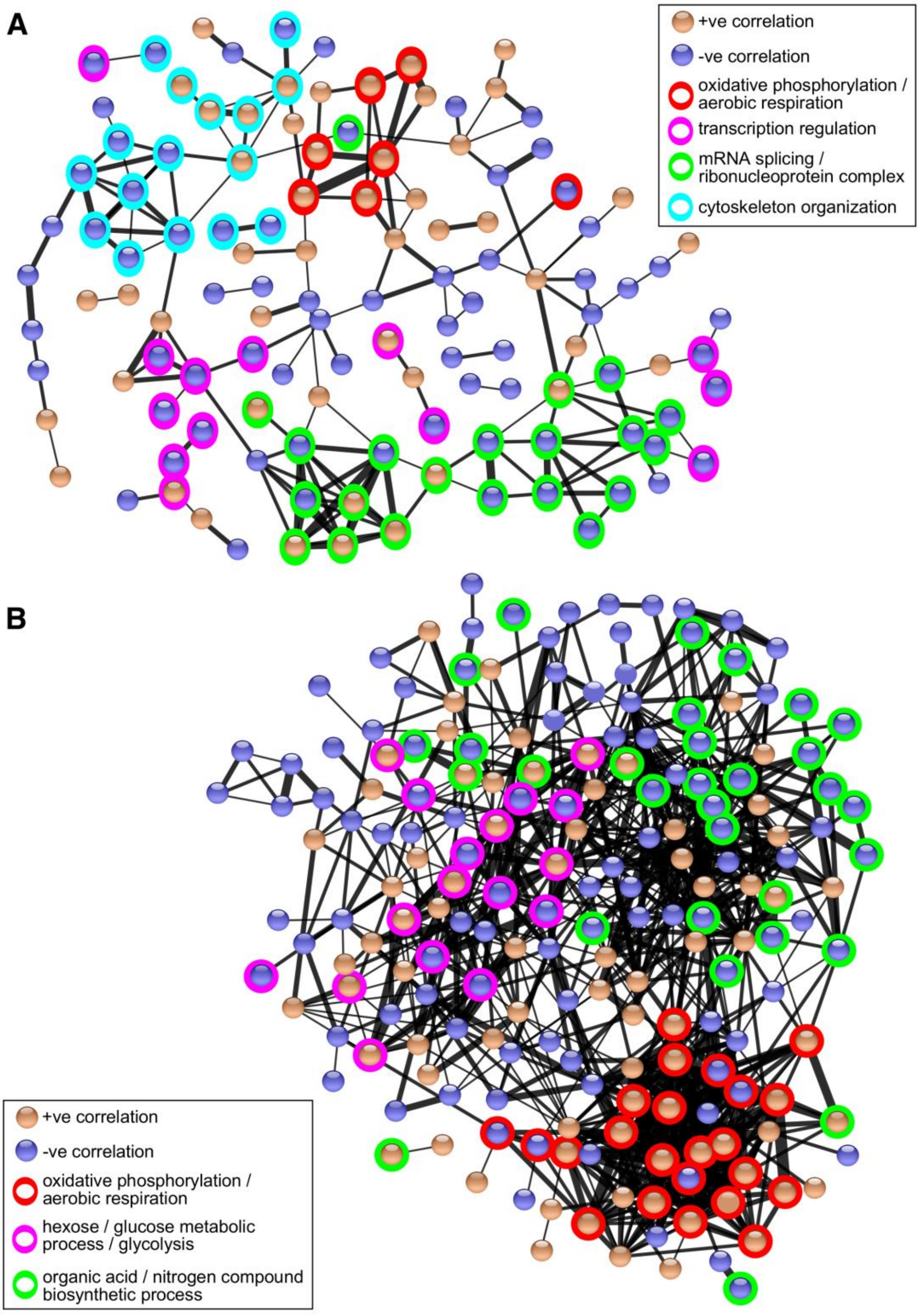

Supplementary Figure 4. Protein-protein interaction network of top hits identified based on (A) transcripts and (B) peptide fragments. The interaction network is based on STRING database (evidence view, high confidence). Genes without interacting partners are omitted. Selected pathways are indicated by colored rings.

SUPPLEMENTARY FIGURE 5

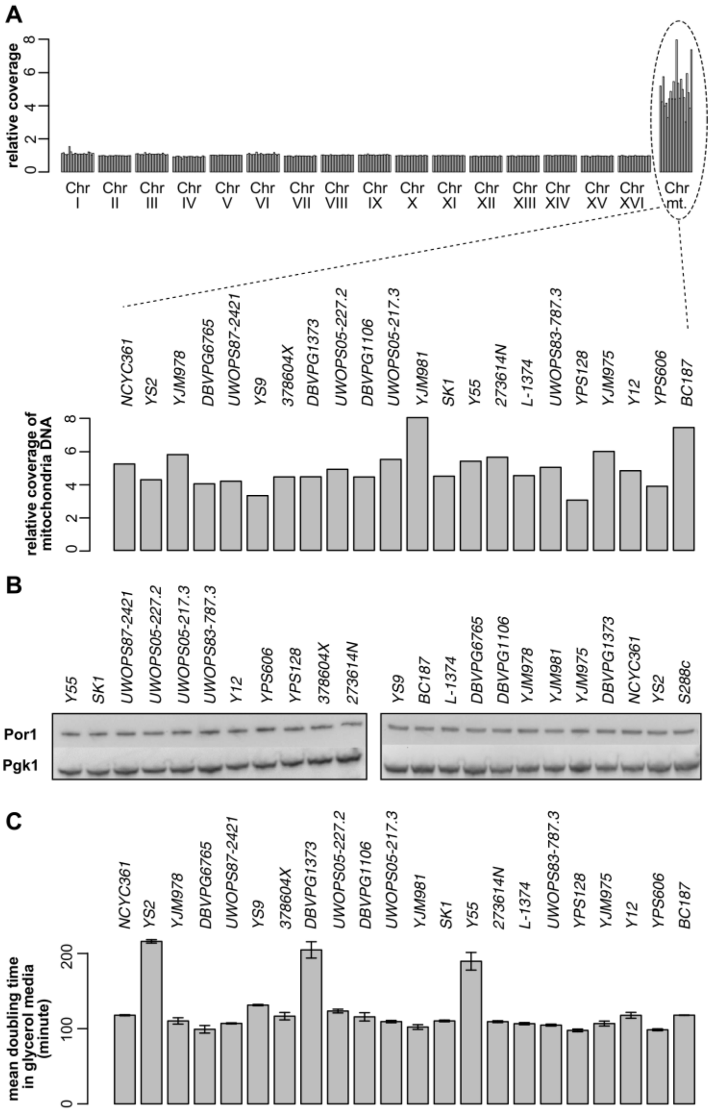

Supplementary Figure 5. Analysis of mitochondria across the strains. (A) Relative coverage of the nuclear and mitochondrial DNA. The upper panel shows the relative coverage for each of the nuclear DNA and mitochondrial DNA (mean coverage across the chromosomes for each strain is set as 1.0). Each bar represents one chromosome in one strain. The strains are ordered by their mean replicative lifespan (see lower panel and Figure 4). The lower panel shows the enlarged view for mitochondrial DNA. **(B) Expression of a mitochondrial protein across the strains.** Western blotting shows that the strains contain similar expression of mitochondrial porin Por1 (voltage-dependent anion channel). Pgk1 is used as internal loading control**. (C) Mean doubling time in glycerol media.** The error bars indicate standard error.

SUPPLEMENTARY FIGURE 6

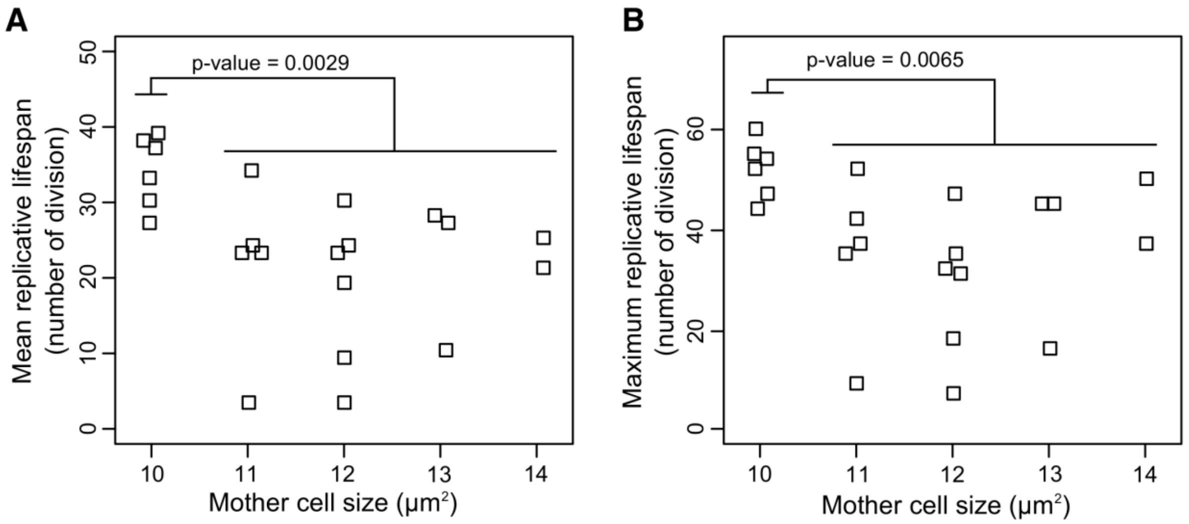

Supplementary Figure 6. Variation of (A) mean replicative lifespan and (B) maximum replicative lifespan according to mother cell size. p-values refer to ANOVA test comparing 10 μm^2 mother cells against 11-14 μm^2 mother cells. Overlapping points are jittered for visualization.
